# Event-Based Safety Indicator Analysis of a Surround View Monitoring (SVM)-Based Pedestrian Detection System Using Real-World Urban Data

**DOI:** 10.3390/s26154931

**Published:** 2026-08-04

**Authors:** Hyeon-Suk Jeong, Jong-Hoon Kim, Hee-Jun Shin, Seong-Pyo Cho

**Affiliations:** 1 Department of Highway & Transportation Research, Korea Institute of Civil Engineering and Building Technology, Goyang-si 10223, Republic of Korea; jhs2204@kict.re.kr (H.-S.J.); jayjay1117@kict.re.kr (H.-J.S.); 2 Hbrain Co., Ltd., Seongnam-si 13105, Republic of Korea; spcho@hbrain.co.kr

**Keywords:** surround view monitoring (SVM), pedestrian detection system, surrogate safety measures (SSM), time to collision (TTC), detection-to-braking time (DBT)

## Abstract

**Highlights:**

**What are the main findings?**

- Real-world urban driving data showed that SVM-defined danger zones were associated with lower time to collision (TTC) and higher TIT/TET values than warning zones, indicating differences in event-level risk characteristics.
- The 95th percentile total jerk showed a decreasing trend in the danger zone over four driving days, but this finding remains exploratory.

**What are the implications of the main findings?**

- SVM-based pedestrian detection systems can be assessed using surrogate safety measures and vehicle-control indicators in addition to conventional detection accuracy.
- The proposed event-based analysis provides a practical framework for examining the relationship between SVM risk-zone classification and safety-related driving indicators under real-world urban conditions.

**Abstract:**

Pedestrian safety in urban areas remains a critical concern, especially for vehicles with large blind spots. This study examined associations between surround view monitoring (SVM)-defined risk zones and safety-related indicators using real-world driving data from school zones and pedestrian crash-prone areas in Seoul, South Korea. Vehicle on-board diagnostics, RTK positioning, SVM object-detection outputs, and video data were collected, and vehicle–vulnerable road user interactions were reconstructed as events. Detection-to-braking time (DBT), time to collision (TTC), time-exposed TTC (TET), time-integrated TTC (TIT), TIT/TET, and jerk were used to compare danger and warning zones. The danger zone showed lower estimated TTC and higher TIT/TET values than the warning zone, indicating shorter time margins and higher average risk intensity during below-threshold TTC intervals. The warning zone showed higher longitudinal jerk, suggesting repeated vehicle-control adjustments during longer events. In the four-day repeated-driving dataset, the 95th percentile total jerk in the danger zone decreased over time; however, this uncontrolled trend was interpreted as exploratory rather than causal. These findings suggest that SVM-defined risk zones are associated with differences in event-level risk and vehicle dynamic indicators, supporting their potential use in event-based safety assessment.

## 1. Introduction

As part of ongoing efforts to improve pedestrian safety, the Government of the Republic of Korea has implemented various policies to reduce pedestrian traffic crashes, including strengthened enforcement in school zones, mandatory complete stops before right turns, and the expansion of pedestrian-priority roads. The deployment of in-vehicle pedestrian detection and warning systems has also continued to increase. Nevertheless, casualties caused by vehicle–pedestrian crashes remain high; crashes involving high-front vehicles and large commercial vehicles have been reported to be particularly severe. Owing to their structural characteristics, high-front vehicles and large commercial vehicles have extensive visual blind spots, which increase the risk of conflicts with pedestrians in complex urban traffic environments, such as right-turn sections at intersections and crosswalks. Crashes often occur when drivers experience delays in situational awareness or fail to detect pedestrians, posing a greater risk to vulnerable road users (VRUs), including children and older adults. Although the blind-spot safety problem is particularly critical for high-front and large commercial vehicles, the present study used a high-bodied passenger minivan as an experimental platform; therefore, the findings should be interpreted as event-based observations under real-world urban vehicle–VRU interaction conditions rather than as direct evidence generalizable to all large commercial vehicles.

Pedestrian detection technologies have mainly been developed around advanced driver assistance systems (ADAS), such as forward collision warning (FCW) and blind spot detection. However, most of these technologies were developed primarily for passenger vehicles and do not sufficiently account for the structural blind-spot characteristics of large commercial vehicles. In addition, previous studies have largely focused on technical evaluations such as sensor recognition performance and object detection accuracy, whereas relatively few studies have empirically examined changes in driver behavior and safety-related effects in real-world urban driving environments.

Moreover, most studies on pedestrian detection systems have been conducted using driving simulators or limited test environments, making it difficult to fully capture the diverse interaction scenarios that occur in actual traffic environments. In urban areas, various hazardous situations arise, including illegal parking, complex pedestrian flows, and the simultaneous entry of right-turning vehicles and pedestrians. In such environments, examining object detection performance in addition to changes in perception, response, and risk-avoidance behavior of the driver is crucial. Therefore, recent studies have increasingly used surrogate safety measures (SSMs), such as time to collision (TTC), time-integrated TTC (TIT), and time-exposed TTC (TET), to quantitatively assess risk during real-world driving.

This study does not aim to establish the causal safety effect of the SVM-based pedestrian detection system because all data were collected under system-active conditions, and no baseline (no-warning) condition was available. Instead, this study examines whether SVM-defined risk-zone classification is associated with differences in event-level safety-related indicators during real-world urban vehicle–VRU interactions. To this end, real-world driving data were collected from school zones and pedestrian crash-prone areas, and vehicle–VRU interactions were reconstructed as event units. Detection-to-braking time, TTC, TET, TIT, TIT/TET, and jerk were used to characterize differences in braking response latency, collision-risk exposure, and vehicle dynamic behavior between the danger and warning zones.

## 2. Literature Review

### 2.1. Trends in Blind-Spot Safety Technologies for Large Vehicles

Large commercial vehicles have more extensive visual blind spots than passenger vehicles owing to their greater heights and longer bodies. These blind spots have been identified as major contributing factors to vehicle–pedestrian crashes at urban intersections, particularly during right-turn maneuvers. In particular, the side areas and near-front zones of large vehicles are difficult for drivers to observe directly, increasing the risk of vehicle conflicts with pedestrians and cyclists [1]. Conventional visibility-support methods based on side and wide-angle mirrors have limitations in sufficiently eliminating blind spots because of restricted viewing angles and distance distortion. Therefore, various driver assistance systems have been investigated to improve the situational awareness of drivers.

Recent studies have actively examined SVM and camera-based pedestrian detection systems as complementary technologies for mitigating blind-spot problems. Lin [2] conducted an empirical study on large buses and reported that side cameras and hybrid mirror systems improve object detection rates compared to conventional mirror-based systems, suggesting their potential to enhance drivers’ awareness of blind spots. In addition, active safety systems such as ADAS have evolved beyond simple visual information provision by delivering warnings to drivers when hazardous situations occur, thereby supporting pedestrian protection. Kovaceva et al. [3] conducted a virtual safety assessment of vehicle–cyclist overtaking scenarios and reported that collision warning systems are associated with shorter driver response times and a reduced occurrence of hazardous situations.

International vehicle safety assessment programs continue to strengthen evaluation protocols to protect pedestrians and VRUs. The Euro New Car Assessment Programme (Euro NCAP) expanded its assessment of autonomous emergency braking and VRU-related functions while strengthening active safety evaluation protocols that consider crossing scenarios, occluding pedestrians, and weather variations [4]. This trend indicates a shift from post-crash injury mitigation to crash prevention through active safety technologies. The Euro NCAP also expanded its safety rating framework for medium and heavy commercial vehicles, emphasizing the need for collision-avoidance technologies that address pedestrian crash risks in urban intersections and low-speed driving environments [4].

Existing sensor technologies have distinct advantages and limitations. RGB camera-based systems can provide rich visual information; however, their recognition performance may degrade under adverse conditions, such as nighttime, rain, or fog [5]. Radar sensors are good at measuring distance and relative speed; however, they have limitations in recognizing the shape of near-field objects [6,7]. LiDAR sensors can provide accurate distance measurements; however, high system costs and near-field shadow zones caused by vehicle structures remain major challenges [8]. Therefore, sensor-fusion-based approaches that combine RGB cameras, infrared cameras, radar, LiDAR, and ultrasonic sensors have been increasingly investigated to achieve robust pedestrian detection performance under varying environmental conditions [5,9].

### 2.2. Definition of Surrogate Safety Measures and Previous Studies

As actual crash data occur relatively infrequently, securing sufficient statistical significance using crash records alone is often difficult. SSMs are widely used to quantify hazardous situations that occur during driving. Representative SSMs include TTC, TIT, and TET [10,11,12].

TTC, first introduced by Hayward in 1972, refers to the time remaining before two objects collide if they maintain their current speeds [11]. It has been widely used as a risk-assessment criterion in ADAS. In longitudinal car-following contexts, TTC is commonly expressed using Equation (1).

(1) $$TTC=\frac{\left[x_{n-1}\left(t\right)-x_{n}\left(t\right)-s_{n-1}\right]}{\left[v_{n}\left(t\right)-v_{n-1}\left(t\right)\right]}$$

where $v_{n-1}\left(t\right)$ is the speed of the leading vehicle, $v_{n}\left(t\right)$ is the speed of the following vehicle, $x_{n-1}\left(t\right)$ is the position of the leading vehicle, and $x_{n}\left(t\right)$ is the position of the following vehicle at time t; $s_{n-1}$ is the length of the leading vehicle. Tak et al. [10] emphasized that TTC is highly correlated with human driving behavior in ADAS evaluations; however, they also noted that it should be used together with other indicators in complex interaction scenarios. This equation is presented as a conventional TTC formulation used in previous car-following studies. In this study, the TTC concept was extended to vehicle–VRU interactions to estimate the event-level time margin between the experimental vehicle and detected vulnerable road users.

TIT is an indicator that accumulates the level of risk exposure over the period during which the TTC remains below a predefined threshold. Whereas TTC represents the instantaneous collision risk, TIT can reflect both the duration and severity of hazardous situations. Minderhoud and Bovy [12] defined TIT as the time integral of the difference between the TTC threshold and actual TTC value. Therefore, TIT has been used as a representative cumulative risk indicator that complements the limitations of TTC-based measures.

(2) $$TIT=\int_{t\in TTC<{TTC}^{*}}\left({TTC}^{*}-TTC\left(t\right)\right)dt$$

where ${TTC}^{*}$ denotes the predefined TTC threshold, and risk exposure is accumulated only during the period in which TTC is lower than the threshold. TET refers to the total duration for which TTC remains below a predefined threshold and represents the duration of a hazardous situation. In other words, a higher TET value indicates that the driver was exposed to a critical risk level for a longer period. In contrast, TIT reflects not only the duration of risk exposure but also the intensity of the risk. Therefore, a higher TIT value indicates that a hazardous situation persisted for a longer duration or involved a higher level of collision risk. Recently, TET and TIT have been increasingly used together in urban pedestrian conflict and complex traffic environment analyses because they can capture risk-exposure characteristics more effectively than the TTC alone.

Studies have now advanced beyond conventional one-dimensional indicators to better reflect the complex conflicts in urban environments. Xu et al. [13] proposed a two-dimensional SSM that considers the lateral collision risk and uncertainty, providing a more refined approach to risk estimation in urban driving environments.

### 2.3. Previous Studies on Driver Perception–Reaction Time

PRT refers to the time interval between the moment when a driver perceives a hazard and the moment when the driver initiates an evasive action, such as braking. It is also widely used as a key indicator for explaining changes in driver responses and safety-related effects when evaluating driver assistance and safety systems.

In general, the average PRT of drivers under normal driving conditions is known to be approximately 1.25–1.5 s; however, it may vary depending on the predictability of the hazardous situation, the driver’s attention level, and the provision of warning systems [14]. In roadway design, a PRT of 2.5 s is commonly assumed, based on the stopping sight distance criteria in the AASHTO Green Book, as introduced in the FHWA speed concepts document, to apply conservative safety standards [15]. This value can be interpreted as a conservative safety criterion that accounts for various driver characteristics and unexpected hazardous situations, rather than as a typical average response time.

Previous studies have reported that unexpected situations, such as the sudden appearance of a pedestrian, can produce greater variability in driver response time than ordinary car-following situations (Table 1) [16]. In addition, when warning systems such as FCW and pedestrian-to-vehicle (P2V) warnings are provided, drivers perceive hazards and initiate braking earlier, showing a tendency toward shorter average braking response times [17,18]. Although repeated experiences with warning systems may influence driver behavioral adaptation and learning effects, system reliability and warning presentation methods have also been suggested as important factors affecting changes in driver response characteristics [17,19].

### 2.4. Research Contribution

Previous studies have several limitations. First, most studies have been conducted using driving simulators or controlled proving-ground environments [2,3], which limits their ability to reflect irregular pedestrian flows and complex environmental factors in real-world urban areas. Second, previous studies have mainly focused on technical evaluations, such as object detection accuracy and sensor performance. Consequently, relatively few studies have analyzed the effect of pedestrian detection systems on drivers’ hazard perception and behavioral changes in real-world driving environments.

Previous studies on commercial vehicle safety systems primarily focused on individual sensor-based object detection or warning-function evaluations. Although SVM systems based on multichannel camera-driven 360° image composition have recently been commercialized, mainly for passenger vehicles, studies using real-world driving data to examine the influence of such systems on drivers’ hazard perception and safe driving behavior in urban environments remain limited.

Therefore, this study contributes to the literature by providing an event-based empirical analysis of the association between SVM risk-zone classification and safety-related indicators in real-world urban vehicle–VRU interactions. Rather than validating the causal effect of the system, this study focuses on whether SVM-defined danger and warning zones correspond to differences in detection-to-braking time, TTC, TET, TIT, TIT/TET, and jerk indicators. This approach extends previous sensor-performance-oriented evaluations by incorporating driver response latency and vehicle dynamic behavior under real-world driving conditions.

This study was not designed as a comparative performance evaluation of different detection or warning methods. Instead, it focused on examining whether SVM-generated risk-zone labels corresponded to differences in event-level safety-related indicators under real-world urban driving conditions.

## 3. Methodology

### 3.1. Configuration of the SVM-Based Pedestrian Detection System

**Figure 1:**
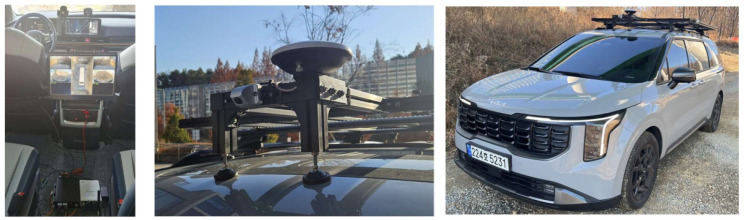
Experimental vehicle setup for real-world driving data collection.

A multichannel SVM-based pedestrian detection system was installed in an experimental vehicle to analyze driver behavioral characteristics and risk-exposure levels under real-world urban driving conditions (Figure 1). A Kia Carnival was used as the experimental vehicle because it has a relatively high vehicle height and a wide body structure and is frequently operated in low-speed urban environments where vehicle–pedestrian interactions occur. Although the Kia Carnival is not a heavy commercial vehicle, it was selected as a practical experimental platform for examining near-field vehicle–VRU interactions around a high-bodied passenger vehicle. Therefore, this study aimed to analyze the association between an SVM-based pedestrian detection system and event-level safety-related indicators, rather than to validate the sensor performance of a specific vehicle model or to represent the full blind-spot characteristics of large commercial vehicles.

A six-channel multimodal sensor network was installed in the experimental vehicle. The system comprised four SVM cameras for a 360° surrounding environment perception around the vehicle and two RGB + IR cameras were installed to support pedestrian recognition under varying lighting conditions. The collected image data were processed in real-time using an edge-AI-based processing unit integrated into an in-vehicle terminal. The system was configured to recognize pedestrians and VRUs and provide warning information to the driver.

In addition, an RTK-based precision positioning system, EDGE INS (U3), was installed to acquire vehicle location and driving trajectory information. This system collected GNSS-based positioning data at a frequency of 1 Hz and stored it in synchronization with the driving log data generated during vehicle operation. This configuration enabled the integrated management of vehicle location, driver behavior, and object detection information.

The data collected in this study comprised vehicle on-board diagnostics (OBD) data, RTK-based positioning, SVM object detection, and video data. The OBD data included the vehicle speed, steering angle, braking signal, longitudinal acceleration, and lateral acceleration, which were used to calculate the driver response time and driving stability indicators. The SVM detection data included object types, such as pedestrians, motorcycles, bicycles, and personal mobility devices, as well as warning areas, namely, the warning and danger zones. These data were used to define hazardous events during real-world driving scenarios.

The SVM system provided predefined distance-based risk-zone labels for detected objects. In the system configuration used in this study, objects located within 0≤d<3.0 m from the vehicle were classified as the danger zone and displayed using a red warning signal, whereas objects located within 3.0≤d<5.0 m were classified as the warning zone and displayed using a yellow caution signal. Here, d denotes the estimated distance between the vehicle and the detected object. The warning display position was determined according to the detected object direction, such as the front, rear, left, or right side of the vehicle. These zones were treated as system-defined risk categories rather than externally validated ground-truth risk classes. Therefore, this study did not aim to validate the zone-classification criteria themselves, but examined whether these predefined SVM risk-zone labels were associated with differences in event-level safety-related indicators.

**Figure 2:**
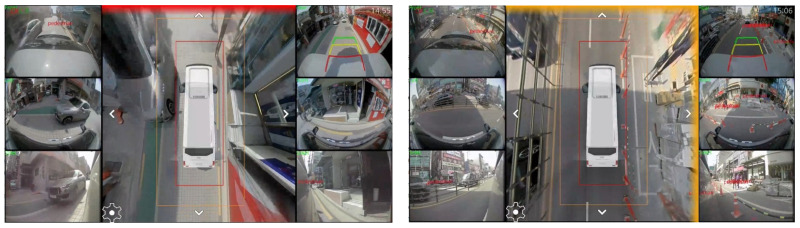
Example of an SVM system operator screen showing predefined distance-based warning and danger zones around the experimental vehicle.

Figure 1 shows the configuration and installation status of the SVM-based pedestrian detection system applied to the experimental vehicle. An SVM display terminal was installed inside the vehicle to provide information to the driver. In addition, SVM cameras for 360° surrounding environment perception, RGB + IR cameras to support pedestrian recognition under adverse environmental conditions, and an RTK-based positioning device were installed on the roof of the vehicle. Figure 2 shows an example of the SVM operator display screen, illustrating the predefined danger and warning zone described above as they appear during real-time operation.

### 3.2. Real-World Driving Data Collection Environment

Empirical test sites were selected from school zones and pedestrian crash-prone areas to examine the safety-related effects of the SVM-based pedestrian detection system under real-world urban driving conditions. To identify candidate test sites, pedestrian traffic fatality statistics in Seoul over the most recent 5-year period (2021–2025) were reviewed (Table 2). These statistics were used as baseline data to objectively assess the level of pedestrian crash risk in each district.

In selecting the final empirical test sites, several factors were considered, including the distribution of school zones, presence of urban intersections and right-turn environments, and the likelihood of vehicle–pedestrian conflict situations. Gangseo-gu, Dongjak-gu, and Gangnam-gu were selected as the final empirical test areas, based on a comprehensive review of their potential to capture diverse hazardous events.

An event-based empirical analysis was conducted in Gangseo-gu on 29 April 2026, and in Dongjak-gu on 30 April 2026. These datasets were used as the main datasets for analyzing driver response characteristics and risk-exposure levels under various vehicle–pedestrian interaction scenarios. In addition, time-series data were collected in Gangnam-gu from 11 to 14 May 2026, to examine trends in safety indicators during the utilization of the SVM system.

During the empirical tests, vehicle OBD, RTK-based positioning, SVM-based object detection, and video data were collected in a synchronized manner. The OBD data included the vehicle speed, steering angle, longitudinal and lateral accelerations, and braking signals, whereas the RTK-based positioning data recorded the location and driving trajectory of the experimental vehicle. SVM object detection and video data were used to define vehicle–pedestrian interaction situations and hazardous events. The empirical driving tests were conducted by a single licensed driver. Therefore, the findings should be interpreted as event-level observations from a limited real-world driving dataset rather than as generalizable driver-population-level effects.

### 3.3. Event Definition and Data Preprocessing

Vehicle OBD, RTK-based positioning, SVM-based object detection, and video data were integrated to analyze pedestrian and VRU interaction events during real-world driving. All the collected data were synchronized based on timestamp information; hazardous situations were defined and analyzed at the event level. Vehicle OBD data were used to analyze driver control characteristics and vehicle dynamic behavior, whereas RTK-based positioning data were used to examine vehicle location and driving trajectories. In addition, SVM object detection data included object type and risk-zone information and were used to define hazardous events and analyze vehicle–pedestrian interaction situations. The data structures used in this study are listed in Table 3.

Although the SVM system implemented real-time VRU detection, the analysis dataset used in this study consisted of frame-level detection logs and did not include a stable tracking ID for continuously identifying the same object across all frames. Therefore, a rule-based event reconstruction procedure was applied. Detection records were grouped as one vehicle–VRU interaction event when they had the same object class and were detected within a 2 s temporal gap either by the same camera or by spatially adjacent cameras according to the predefined camera-view configuration of the SVM system. Each camera number corresponded to a fixed viewing direction around the vehicle, such as the front, rear, left, or right side. Therefore, when a detected VRU appeared sequentially in adjacent camera views within a short time interval, for example, from the right-side camera to the rear camera, the detections were treated as part of the same interaction event if the object class and risk-zone continuity were consistent. This procedure was intended to reduce artificial event splitting caused by ego-vehicle movement, temporary occlusion, frame drops, or transitions between camera fields of view.

Because this approach is a rule-based event reconstruction method rather than a trajectory-level object-tracking algorithm, it cannot fully eliminate the possibility of merging different VRUs or splitting a continuous interaction into multiple events. This procedure also cannot fully eliminate the possibility of concurrent duplicate detections when the same VRU is captured by overlapping camera views. Accordingly, the analysis focused on aggregated differences between SVM-defined danger and warning zones rather than on precise trajectory-level risk estimation for each individual pedestrian.

In addition, when the vehicle was stopped or moving at an extremely low speed, the relative speed between the vehicle and the pedestrian was close to zero, which made it difficult to meaningfully calculate indicators such as TTC. Therefore, only events with an average vehicle speed of 5 km/h or higher during the event interval were classified as driving interactions and selected as the final analysis targets.

For each driving event identified using the aforementioned procedure, the first detection time within the cluster was defined as the initial object recognition time; the time at which the brake_pedal_switch was activated in the OBD data was defined as the driver’s braking response time. The final analysis dataset was constructed through duplicate event removal, missing value removal, and time synchronization.

### 3.4. Definition of Pedestrian Safety Performance Indicators

In this study, pedestrian safety performance indicators were established to examine safety-related event characteristics of an SVM-based pedestrian detection system by reflecting braking-response latency, risk-exposure levels, vehicle–pedestrian interaction safety and driving stability. The indicators comprised Detection-to-Braking Time (DBT), TTC, TET, TIT, and jerk; their calculation methods and input data are summarized in Table 4.

DBT was calculated only for events in which brake activation occurred at least once during the reconstructed event and the brake pedal was not already activated at the event onset; events with no brake activation, or events that had already started under braking, were excluded because the time from initial SVM detection to braking initiation could not be meaningfully defined for these cases. In addition, among these valid braking events, events for which the resulting DBT value exceeded the response-time range reported in previous studies [15] were further excluded, to reduce the influence of atypical, delayed, or unrelated braking behavior. Therefore, the DBT results were interpreted as conditional braking-response observations restricted to events with a clearly identifiable and plausible braking onset.

Although the conventional TTC formulation is often presented for longitudinal car-following situations, the same concept can be generalized to vehicle–VRU interactions by defining TTC as the estimated separation distance divided by the approaching relative speed. In this study, d (t) denotes the estimated distance between the experimental vehicle and the detected VRU, and $v_{rel}\left(t\right)$ denotes the approaching relative speed derived from the temporal change in the estimated distance. Therefore, TTC for each frame was calculated as $TTC\left(t\right)=d\left(t\right)/v_{rel}\left(t\right)$ only when $v_{rel}\left(t\right)>0$.

TTC was calculated based on the estimated object distance and relative speed in each frame. Object distance was estimated from the SVM detection output using camera-based geometric approximation, and relative speed was derived from the temporal change in estimated distance within each reconstructed event. Specifically, the lower vertical coordinate of the detected bounding box was used as an approximate ground-contact point of the VRU, and the distance from the vehicle was estimated based on the camera installation geometry and ground-plane assumption. The relative speed used for TTC calculation was not estimated from an independently tracked pedestrian trajectory but was derived as the approaching speed from the temporal change in the estimated vehicle–VRU distance within each reconstructed event. Only periods in which the vehicle was approaching the object, i.e., when the relative speed was positive, were included in the TTC analysis. In addition, TTC values greater than 15 s were treated as outside the predefined valid analysis range and were excluded from the event-level TTC analysis. This upper bound was used as a data-screening criterion to reduce the influence of long-horizon interactions and unstable TTC estimates caused by very small relative-speed values; it was not used as the risk threshold for TET or TIT calculation.

Therefore, only positive TTC values of 15 s or less were retained as valid TTC observations. For event-level analysis, the 5th percentile value of the TTC distribution within each event was defined as the representative TTC of that event. This approach was used to reduce the influence of distance-estimation errors and instantaneous noise that may occur in a single frame, while still reflecting relatively high-risk intervals within the event. Subsequently, the mean and median values for the warning and danger zones were calculated by aggregating the representative TTC values of the events within each zone. The TTC threshold was set to 3 s, following a commonly adopted criterion in previous studies that jointly used TTC, TET, and TIT [20]. Because TTC-related indicators were derived from camera-based distance estimates rather than directly measured pedestrian trajectories, they were interpreted as approximate event-level surrogate safety indicators.

Jerk was defined as the rate of change in acceleration over time and was used to quantify abrupt vehicle movements, such as sudden acceleration, braking, and steering. Longitudinal jerk and lateral jerk were calculated from the time derivatives of longitudinal and lateral acceleration obtained from the OBD data. Specifically, $j_{long}\left(t_{k}\right)$ and $j_{lat}\left(t_{k}\right)$ were calculated as the change in acceleration between consecutive timestamps divided by the corresponding time interval. Total jerk was then calculated as $j_{total}\left(t_{k}\right)=\sqrt{j_{long}{\left(t_{k}\right)}^{2}+j_{lat}{\left(t_{k}\right)}^{2}}$. In this study, the mean jerk of each event was used to evaluate the overall intensity of vehicle-control variation, whereas the 95th percentile jerk was used to assess high-intensity instantaneous vehicle dynamic responses within the event.

### 3.5. Statistical Analysis Methods

The event-based analysis was conducted through sequential steps consisting of data synchronization, rule-based event reconstruction, driving-event selection, safety-indicator calculation, and statistical comparison between SVM-defined danger and warning zones. Because several preprocessing criteria and threshold values were applied during this process, they are summarized in Table 5 to improve the reproducibility and clarity of the analysis.

Statistical analyses were conducted to examine whether driver response, collision risk, and vehicle dynamic behavior differed between risk zones in real-world driving environments in which the SVM-based pedestrian detection system was provided. First, the differences in pedestrian safety indicators between the danger and warning zones were compared. The indicators included DBT, TTC, TET, TIT, TIT/TET, and jerk-related measures. As event-based real-world driving data cannot be assumed to follow a normal distribution, and some indicators may include zero or extreme values, the nonparametric Mann–Whitney U test was applied to compare the two zones.

In addition to statistical significance, effect sizes were calculated to examine the practical magnitude of the observed differences, which were used as a nonparametric effect size measure for two independent groups. Effect sizes were interpreted according to the criteria (Table 6) proposed by Romano et al. [21]. The sign of the Cliff’s delta indicates the relative magnitude of the indicator values between the danger and warning zones; a negative value indicates that the value in the danger zone is lower than that in the warning zone. As multiple statistical tests were conducted across several safety indicators, Holm correction was applied to reduce the possibility of Type I errors owing to multiple comparisons. The statistical significance level was set to 0.05.

Because multiple events were collected from the same experimental vehicle, along limited routes and repeated driving sessions, the event-level observations may not be fully independent. In addition, because events were reconstructed at the zone level, a single continuous vehicle–VRU interaction that transitions between the warning and danger zones could, in principle, contribute observations to both statistical groups, which is a further potential source of non-independence. Therefore, the Mann–Whitney U test was used as a descriptive nonparametric comparison of event-level distributions, and the results were interpreted together with effect sizes rather than based solely on *p*-values. Accordingly, statistical significance was not interpreted as evidence of causal safety effects, but as an indication of distributional differences between SVM-defined danger and warning zones within the collected dataset.

For the repeated-driving data collected in Gangnam-gu, an additional analysis was conducted to examine the possibility of changes in vehicle dynamic behavior associated with repeated exposure to SVM warnings. To this end, the driving day was defined as an ordinal variable from Days 1 to 4; simple linear regression analyses were conducted separately for the danger and warning zones. In the repeated-driving analysis, the 95th percentile total jerk, which represents the high-intensity vehicle dynamic response, was set as the dependent variable, and driving day order (day_no) was used as the explanatory variable to examine the direction of change as the number of repeated-driving days increased.

However, the real-world driving data used in this study were not collected under fully controlled experimental conditions with identical traffic volumes, pedestrian densities, event compositions, and driving environments on different driving days. Therefore, the repeated-driving analysis was interpreted as an exploratory analysis of changes in vehicle dynamic indicators across driving days, rather than as evidence of driver adaptation or a causal effect of repeated SVM warning exposure. The statistical analysis results are presented as the mean, median, 95th percentile, *p*-value, adjusted *p*-value, and effect size for each indicator to examine whether the SVM risk-zone classification is statistically associated with differences in driver response time, collision risk, and vehicle control behavior.

## 4. Results

### 4.1. Descriptive Analysis of Real-World Driving Data

Pedestrian interaction situations were analyzed based on object detection and vehicle dynamic data collected during real-world driving. The scale of driving data collection and object detection characteristics differed across the empirical test sites; hence, the basic characteristics of the collected data were examined prior to the safety analysis.

Table 7 summarizes the data collected from each empirical test site. A total of 295.49 km of driving data were obtained over 15.74 h of real-world driving, and 389,280 object detection records were collected.

**Figure 3:**
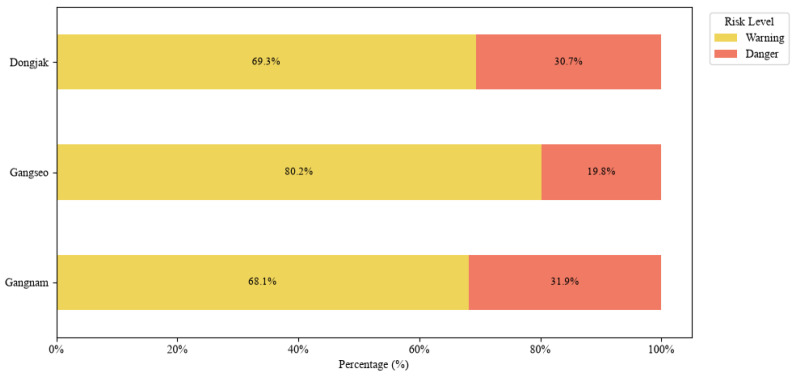
Distribution of warning and danger events by location.

Figure 3 shows the proportions of detections in the warning and danger zones by test sites. At all the test sites, the proportion of detections in the warning zone was higher than that in the danger zone. The proportion of detections in the warning zone ranged from 68.1% to 80.2%, whereas that in the danger zone ranged from 19.8% to 31.9%. In particular, Gangnam-gu and Dongjak-gu showed higher danger-zone proportions (31.9% and 30.7%, respectively), compared with Gangseo-gu (19.8%), indicating that relatively more high-risk interactions were observed in these areas.

Table 8 presents the object detection results by object type and location. Among the 389,280 object detection records, pedestrians accounted for the largest proportion (319,543 records, 82.1%), followed by motorcycles (37,383 records, 9.6%), bicycles (29,010 records, 7.5%), and personal mobility devices (3344 records, 0.9%). This distribution appears to reflect the fact that the empirical tests were conducted mainly in school zones and pedestrian-oriented urban environments.

### 4.2. Analysis of Pedestrian Safety Indicators

Event classification resulted in the identification of 16,436 object interaction events. Among these, 6945 events were classified as driving events, and 9491 events were classified as stopped or low-speed waiting events. Many stopped events were observed during signal waiting and intersection congestion. To ensure the validity of the analysis of driver response behavior and safety indicators, only driving events were used as the final analysis targets. This classification indicates that 389,280 frame-level object detection records were integrated into 16,436 object interaction events based on the clustering criteria defined in Section 3.3.

#### 4.2.1. Detection-to-Braking Time (DBT) Analysis

DBT was calculated to analyze braking-response latency in an environment in which the SVM-based pedestrian detection system was provided. DBT was defined as the time difference between the initial object detection time and the driver’s first braking response time. The analysis was conducted according to the event definition and data preprocessing criteria described in Section 3.4.

By location, the mean DBT was 0.87 s in Gangseo-gu and 1.24 s in Dongjak-gu. The empirical test in Gangseo-gu was conducted mainly in school zones and pedestrian-dense areas, where pedestrian interaction events occurred relatively frequently. In contrast, Dongjak-gu had a lower frequency of hazardous events and a higher average driving speed. These environmental differences may have influenced the differences in response times between the two areas.

By risk zone, the mean DBT in Gangseo-gu was 0.78 s in the danger zone and 0.90 s in the warning zone. In Dongjak-gu, the mean DBT was 1.21 s in the danger zone and 1.25 s in the warning zone. In both areas, shorter response times were observed in the danger zone, indicating the tendency of the driver to initiate braking responses more rapidly in higher-risk situations.

**Figure 4:**
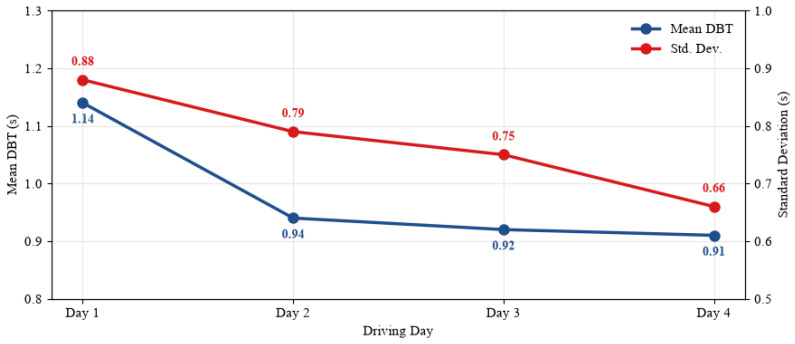
Temporal changes in mean DBT and standard deviation during repeated SVM use.

Using the 4-day repeated-driving data collected in Gangnam-gu, changes in the response time during the utilization of the system were analyzed (Figure 4). The mean DBT gradually decreased from 1.14 s on Day 1 to 0.94 s on Day 2, 0.92 s on Day 3, and 0.91 s on Day 4, corresponding to an approximately 20.2% decrease compared with Day 1. This result shows a decreasing trend in detection-to-braking time across the four repeated driving days. However, because the data were collected under uncontrolled real-world conditions, including variations in traffic volume, pedestrian density, route conditions, and event composition, this trend should be interpreted as exploratory rather than as evidence of driver adaptation caused by repeated SVM warning exposure.

The DBT values derived in this study—0.87 s in Gangseo-gu, 1.24 s in Dongjak-gu, and 1.14 s on Day 1 in Gangnam-gu—were generally shorter than the typical driver response time reported in previous studies (1.25–1.50 s) and the roadway design criterion of 2.5 s [15]. These values indicate that braking responses, when observed after initial SVM detection, occurred within a time range comparable to or shorter than values reported in previous driver response studies. However, because the braking events were not experimentally linked to the SVM warning, these results should be interpreted as detection-to-braking latency rather than direct perception–reaction time.

As an additional sensitivity check, DBT was also examined for the pre-exclusion set of 421 candidate braking events, prior to applying the second-stage exclusion criterion based on the response-time range reported in [15]. Of these, 12 danger-zone events (12.4%) and 58 warning-zone events (17.9%) were excluded, yielding the final 351 observations (85 in the danger zone and 266 in the warning zone), as summarized in the overall statistical comparison presented in Section 4.3. Before this exclusion, the descriptive mean DBT was 1.5326 s in the danger zone and 1.7787 s in the warning zone; after exclusion, the means were 0.9906 s and 1.0128 s, respectively. Although a formal statistical comparison was not repeated on the pre-exclusion set, this pattern suggests that the near-negligible zone-level difference in DBT reported in Section 4.3 is, to some extent, sensitive to this exclusion criterion, and should be interpreted with this caveat.

#### 4.2.2. Time to Collision (TTC), Time Exposed TTC (TET), and Time Integrated TTC (TIT) Analysis

**Figure 5:**
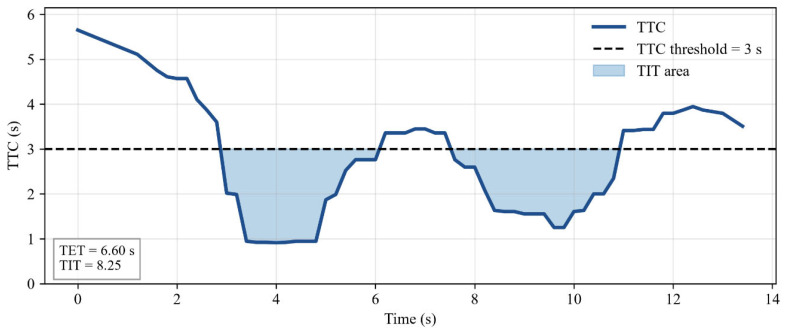
Example of TTC profile and TIT area for a VRU interaction event in Gangseo.

TTC, TET, TIT, and TIT/TET were calculated according to the definitions and procedures described in Section 3.4. These indicators were used to compare event-level time margins, below-threshold exposure duration, cumulative exposure intensity, and average risk intensity between the SVM-defined danger and warning zones. Figure 5 shows a representative example of the TTC profile and the corresponding TIT area for a single VRU interaction event.

Table 9 presents the TTC, TET, and TIT results according to analysis date and risk zone. The results indicate that the mean TTC in the danger zone was shorter than that in the warning zone for all analysis dates. In Gangseo-gu and Dongjak-gu, the mean TTC values in the danger zone were 1.42 s and 1.65 s, respectively, and those in the warning zone were 1.74 s and 1.80 s, respectively. In the repeated-driving data collected in Gangnam-gu, the mean TTC in the danger zone was lower than that in the warning zone from Days 1 to 4. This indicates that the SVM-defined danger zone was associated with lower estimated TTC values than the warning zone, suggesting shorter event-level time margins under the assumptions used for TTC estimation.

In contrast to TTC, TIT reflects both risk intensity and exposure duration; therefore, its pattern does not always correspond to TTC results. The higher mean TIT in the warning zones of Gangseo-gu and Dongjak-gu was mainly associated with longer event durations and larger TET values. However, in Gangnam-gu, the danger zone showed a higher mean TIT from Days 1 to 3, suggesting a more concentrated high-risk exposure within shorter event durations. On Day 4, the higher mean TIT in the warning zone was primarily related to the longer event duration.

The TIT/TET ratio was also examined to complement the interpretation of TIT. TIT/TET represents the average risk intensity during the interval in which TTC remains below the threshold. The results indicate that the TIT/TET in the danger zone was higher than that in the warning zone for all analysis dates. This indicates that, although the duration of risk exposure may be shorter in the danger zone, the risk intensity in the below-threshold interval is higher than that in the warning zone.

Therefore, TTC was interpreted as an indicator of instantaneous collision risk, whereas TET and TIT were interpreted as indicators of the duration and cumulative level of risk exposure below the TTC threshold. Overall, the SVM-defined danger zone showed lower estimated TTC and higher TIT/TET values than the warning zone. These findings suggest that the SVM risk-zone classification was associated with differences in event-level surrogate safety indicators. However, because the TTC-related indicators were derived from estimated object distance and relative speed rather than directly measured pedestrian trajectories, the results should be interpreted as approximate surrogate indicators rather than exact measures of collision risk.

#### 4.2.3. Vehicle Dynamic Response Analysis Based on Jerk

A jerk analysis was conducted to evaluate driver vehicle control behavior under SVM-based warning conditions. Table 10 presents the jerk analysis results by analysis date and risk zone. Overall, the total jerk was higher in the warning zone than in the danger zone. The mean total jerk was higher in the warning zone on most of the analysis dates, although the difference between the two zones was small. In addition, the 95th percentile of total jerk was higher in the warning zone for all analysis dates. This indicates repeated changes in vehicle control, such as deceleration, reacceleration, or steering correction, as warning-zone events persist longer.

A similar pattern was observed for the longitudinal jerk. As the longitudinal jerk is associated with changes in acceleration and braking, these results indicate that the driver adjusted their vehicle speed more continuously in the warning zone. This finding is consistent with the TET and TIT results, in which the risk-exposure duration in the warning zone was relatively longer.

By contrast, the lateral jerk exhibited a pattern different from that of the longitudinal jerk. The mean lateral jerk was higher in the danger zone than in the warning zone for most analysis dates, suggesting that more direct steering responses may have occurred in the danger zone. However, the 95th percentile lateral jerk was higher in the warning zone for all analysis dates. This indicates that immediate steering responses occurred over a short period in the danger zone, whereas intermittent steering corrections occurred during the longer duration of warning-zone events, even when the average level of steering change was not large.

In the repeated-driving data collected in Gangnam-gu, the jerk indicators showed a decreasing trend over time in the descriptive statistics. Comparing Days 1 and 4, the mean total jerk in the danger zone decreased from 0.225 to 0.167, and the 95th percentile total jerk decreased from 0.770 to 0.532. Both longitudinal and lateral jerks also decreased on Day 4 compared with those on Day 1. These changes were consistent with the decreasing DBT trend described in Section 4.2.1. This decreasing trend may indicate changes in vehicle dynamic responses across repeated driving days. However, because traffic conditions, pedestrian exposure, and event severity were not controlled across days, the trend cannot be attributed specifically to repeated SVM warning exposure.

### 4.3. Overall Statistical Analysis of Pedestrian Safety Indicators

Statistical tests were conducted to compare driver response, collision-risk indicators, and vehicle dynamic behavior between the SVM-defined danger and warning zones. Table 11 presents the results of the Mann–Whitney U test and Cliff’s delta analysis for the major safety-related indicators. Because DBT, TTC-related indicators, and jerk-related indicators required different data availability and validity conditions, the statistical comparisons were conducted using the valid analytical observations available for each indicator, and the corresponding number of valid observations (N) for each zone is reported in Table 11. The results indicate that no statistically significant difference exists in DBT between the danger and warning zones. This suggests that braking response time may not be clearly distinguished by risk zone alone, but may also be influenced by event characteristics, driver judgment, and the surrounding driving environment.

Because multiple events were collected from the same vehicle, routes, and driving sessions, the event-level observations may not be fully independent. Therefore, the statistical tests were interpreted primarily as descriptive comparisons of event-level distributions. The adjusted *p*-values were considered together with Cliff’s delta, and greater emphasis was placed on the magnitude and consistency of effect sizes than on statistical significance alone.

Among the collision-risk indicators, TTC was lower in the danger zone than in the warning zone, whereas TIT/TET was higher in the danger zone; however, both differences showed small effect sizes. This suggests that the danger zone was associated with shorter estimated time margins and higher average risk intensity during below-threshold TTC intervals. In contrast, although TET and TIT showed statistically significant differences, their effect sizes were negligible. This suggests that TET and TIT are influenced not only by instantaneous risk but also by event duration; therefore, they have limitations when used alone to explain differences between risk zones.

For the vehicle dynamic behavior indicators, total jerk and longitudinal jerk were higher in the warning zone than in the danger zone. In particular, the 95th percentile total jerk and the 95th percentile longitudinal jerk were higher in the warning zone, with small effect sizes. This suggests repeated deceleration, reacceleration, or speed adjustment during relatively longer warning-zone events. For the mean lateral jerk specifically, the overall pooled comparison in Table 11 shows a slightly higher value in the warning zone, which differs in direction from the location-level pattern reported in Section 4.2.3 (Table 10), where the mean lateral jerk was higher in the danger zone for most individual dates. This difference reflects the fact that the overall statistic in Table 11 is an event-count-weighted aggregate across all events, and warning-zone events were disproportionately concentrated in locations and dates with a smaller danger–warning gap for this indicator; it does not indicate a reversal of the per-location pattern itself. The 95th-percentile lateral jerk pattern, by contrast, is consistent in direction at both the location level and the overall pooled level.

**Figure 6:**
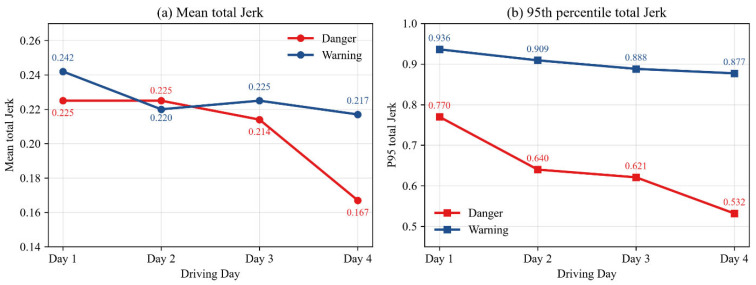
Temporal changes in total jerk during repeated SVM exposure.

For the repeated-driving data collected in Gangnam-gu, the possibility of changes in vehicle dynamic behavior with increasing driving days was further examined. The absolute level of the 95th percentile total jerk in the warning zone generally remained higher than that in the danger zone (Figure 6). However, the trend analysis using the driving day as the explanatory variable showed that the 95th percentile total jerk (Table 12) in the danger zone showed an exploratory decreasing trend as the number of driving days increased. However, because only four uncontrolled driving days were available, this trend should be interpreted as exploratory and not as evidence that repeated SVM use caused the decrease. In contrast, the warning zone showed a slight change in the 95th percentile total jerk, and the trend was not statistically significant.

These results indicate that although the warning zone showed a higher absolute level of vehicle dynamic variation, no clear improvement trend was observed with repeated driving. In contrast, the danger zone showed a gradual decrease in high-intensity vehicle dynamic changes as repeated driving progressed, despite the relatively lower absolute level of the 95th percentile total jerk.

Overall, the danger zone of the SVM system showed a higher collision risk intensity than the warning zone, as indicated by the lower TTC and higher TIT/TET values. In addition, the warning zone was characterized by relatively higher total and longitudinal jerks, suggesting repeated speed adjustments and vehicle control changes. However, most of the effect sizes were negligible or small, indicating that statistically significant differences do not necessarily imply large practical differences. Therefore, the SVM risk-zone classification can be interpreted as being associated with differences in estimated collision-risk indicators and vehicle dynamic behavior during pedestrian interaction events. However, these associations should not be interpreted as causal safety effects of the SVM system because the study did not include a no-warning baseline condition, and the event-level observations were collected under uncontrolled real-world conditions.

## 5. Conclusions

This study examined the association between SVM-defined risk-zone classification and event-level safety-related indicators using real-world urban driving data. Vehicle–pedestrian and vehicle–VRU interaction situations were reconstructed as event units, and detection-to-braking time (DBT), TTC, TET, TIT, TIT/TET, and jerk indicators were used to compare differences between the danger and warning zones. Because all data were collected under system-active conditions, this study was not designed to establish the causal safety effect of the SVM-based pedestrian detection system.

The results showed that the danger zone generally had lower estimated TTC and higher TIT/TET values than the warning zone. This indicates that the danger zone was associated with shorter time margins and higher average risk intensity during intervals in which TTC remained below the threshold. In contrast, the warning zone showed relatively higher total jerk and longitudinal jerk, suggesting repeated vehicle-control adjustments over longer event durations. DBT was descriptively shorter in the danger zone; however, the overall statistical analysis did not identify a clear difference in DBT between the danger and warning zones. This suggests that braking response latency may be influenced not only by SVM risk-zone classification but also by event characteristics, driver judgment, and surrounding traffic conditions. In addition, most effect sizes were negligible or small, indicating that statistically significant differences do not necessarily imply large practical differences.

The repeated-driving data collected in Gangnam-gu showed a decreasing trend in the 95th percentile total jerk in the danger zone across four driving days. However, this result should be interpreted as an exploratory observation rather than evidence of driver adaptation caused by repeated SVM warning exposure, because traffic conditions, pedestrian exposure, route characteristics, and event severity were not experimentally controlled across days.

The main contribution of this study is that it provides an event-based framework for examining how SVM-defined risk zones are associated with surrogate safety indicators and vehicle dynamic responses under real-world urban driving conditions. In contrast to previous studies that mainly relied on simulators, limited test environments, or object-detection-accuracy-based evaluations, this study used field data to characterize vehicle–VRU interaction events and to explore whether system-defined warning levels correspond to differences in estimated TTC-related indicators and vehicle-control behavior. Furthermore, by jointly applying TTC, TET, TIT, TIT/TET, and jerk indicators, this study provides a multidimensional interpretation of instantaneous risk, risk-exposure duration, cumulative risk intensity, and vehicle dynamic variation.

However, this study has several limitations. First, the analysis was based on data collected under conditions in which the SVM system was active, and no direct before-and-after comparison or no-warning control condition was included. Therefore, the findings should not be interpreted as causal evidence that the SVM system improved pedestrian safety. Second, real-world driving data are affected by diverse external factors, such as traffic volume, pedestrian density, driving route, signal operation, and object-type composition, making it difficult to fully control all conditions; because the event-level observations were also collected from a limited number of routes, driving days, and driver conditions, they may not be fully independent, and the statistical significance of event-level comparisons should be interpreted cautiously, with greater emphasis on effect sizes and observed distributional patterns. Third, the SVM object detection data did not include directly measured pedestrian trajectories or absolute pedestrian positions, which may have introduced uncertainty into event reconstruction and TTC calculation. Accordingly, TTC, TET, and TIT were interpreted as approximate event-level surrogate safety indicators rather than exact trajectory-level collision-risk measurements. Fourth, DBT was calculated only for events in which braking responses were observed; therefore, it should be interpreted as conditional brake response latency after initial SVM detection rather than as a direct measure of warning-induced driver perception–reaction time.

Fifth, the experimental vehicle used in this study was a high-bodied passenger minivan rather than a large or heavy commercial vehicle; therefore, the findings should not be directly generalized to all large commercial vehicles. Sixth, although RGB + IR cameras were installed to support pedestrian recognition under varying lighting conditions, the present dataset was not stratified by lighting or weather conditions. Therefore, this study did not evaluate whether the safety indicators differed between daytime, nighttime, clear-weather, or adverse-weather conditions. Seventh, event duration was not statistically controlled in the zone-based comparisons; therefore, the observed differences in TET, TIT, and jerk-related indicators should be interpreted as descriptive event-level associations rather than as independent effects of SVM risk-zone classification.

Future research should collect long-term empirical data across more diverse vehicles, drivers, road environments, and time periods and introduce an experimental design that compares conditions with and without SVM-based warning information. In addition, applying sensor-fusion technologies capable of more precisely tracking the absolute positions and trajectories of pedestrians and VRUs would enable more refined risk analyses, including PET and two-dimensional surrogate safety measures. Furthermore, integrated driver behavior analysis that considers braking, steering, acceleration, deceleration, warning acceptance, and system reliability would allow a more comprehensive assessment of SVM-based pedestrian detection systems under real-world urban conditions.

This study presents an empirical case of event-based safety indicator analysis for an SVM-based pedestrian detection system under real-world urban driving conditions. The findings suggest that SVM-defined risk zones may be useful for characterizing vehicle–VRU interaction events, while also highlighting the need for controlled baseline comparisons, improved object tracking, and more rigorous experimental designs in future evaluations.

## Figures and Tables

**Table 1:** Driver perception–reaction time (PRT) based on previous studies.

Study	Condition	Reaction Time	Implication
Green [14]	Predictable situation Unexpected, general situation Sudden situation	0.70–0.75 s	PRT criteria for general and sudden situations
		1.25 s	
		1.5 s	
FHWA [15]	Stopping sight distance design criterion	2.5 s	Conservative safety judgment criterion
Han et al. [16]	Simulation of child jaywalking	0.24–1.72 s	Reference for a sudden pedestrian appearance
Winkler et al. [17]	Repeated general warnings (T1: no warning to T3: repeated warning)	Scenario 1: 2.6 s → 1.3 s	Evidence of repeated use and learning effects
		Scenario 2: 1.2 s → 0.3 s	
	Repeated emergency warnings (T1: no warning to T3: repeated warning)	Scenario 3: 1.6 s → 0.8 s	
		Scenario 4: 1.0 s → 0.7 s	
Wu et al. [18]	FCW	1.60 s → 1.23 s	Evidence of warning-system effects on brake reaction time
	P2V warning	1.07 s → 0.03 s	Simulator evidence that P2V warnings can shorten brake reaction time by enabling advanced brake preparation
Reinmueller & Steinhauser [19]	Adaptive FCW and reliability conditions	Condition-specific effects	Limitations related to the warning reliability and acceptance

**Table 2:** Pedestrian traffic fatalities by district in Seoul from 2021 to 2025.

Rank	District	2021	2022	2023	2024	2025	Total
1	Songpa-gu	3	3	6	13	10	35
2	Gangseo-gu	8	9	4	6	7	34
3	Gangnam-gu	6	7	5	5	7	30
4	Mapo-gu	6	7	5	5	6	29
5	Dongjak-gu	8	5	5	2	7	27

**Table 3:** Structure of the empirical database.

Table Name	Table Description	Column	Column Description	Data Type
OBD_data	Vehicle OBD data	id	Experiment session ID	INT
		timestamp	Timestamp	DATETIME
		vehicle_id	Vehicle identifier	VARCHAR
		location	Test site	NVARCHAR
		yaw_rate	Yaw rate	FLOAT
		accel_pedal_position	Accel pedal position (%)	FLOAT
		brake_pedal_switch	Brake switch status	BIT
		lateral_acceleration	Lateral acceleration	FLOAT
		longitudinal_acceleration	Longitudinal acceleration	FLOAT
		vehicle_speed	Vehicle speed	FLOAT
		steering_wheel_angle	Steering wheel angle	FLOAT
vehicle_location	RTK-based positioning data	id	Experiment session ID	INT
		timestamp	Timestamp	DATETIME
		vehicle_id	Vehicle identifier	VARCHAR
		location	Test site	NVARCHAR
		latitude	Vehicle latitude	DECIMAL
		longitude	Vehicle longitude	DECIMAL
detection_data	SVM object detection data	id	Experiment session ID	INT
		timestamp	Timestamp	DATETIME
		vehicle_id	Vehicle identifier	VARCHAR
		location	Test site	NVARCHAR
		camera_no	Camera number	INT
		x1	Upper-left x-coordinate of BBOX	INT
		y1	Upper-left y-coordinate of BBOX	INT
		x2	Lower-right x-coordinate of BBOX	INT
		y2	Lower-right y-coordinate of BBOX	INT
		class_id	Object class ID	INT
		class_name	Object type	NVARCHAR
		zone	Warning information zone	VARCHAR

**Table 4:** Definition of safety performance indicators.

Indicator	Definition	Input Variables	Calculation Formula	
DBT	Driver response time after object detection	timestamp, detection.zone, obd.brake_pedal_switch	$t_{brake}-t_{warning}$	
			Here,	$t_{brake}$: time of brake activation
				$t_{warning}$: time of initial hazardous object detection
TTC	Remaining time to collision between the vehicle and pedestrian	timestamp, detection. camera_no, detection.zone, detection.bbox	$d\left(t\right)/v_{rel}\left(t\right),{\;\;\;\;\;v}_{rel}\left(t\right)>0$	
			Here,	d: estimated distance between the vehicle and the detected VRU
				$v_{rel}$: approaching relative speed derived from the temporal change in estimated distance
TET	Duration of risk exposure below the TTC threshold	TTC	$\int_{t_{1}}^{t_{2}}I\left(TTC\left(t\right)<{TTC}^{*}\right)dt$ $I\left(TTC\left(t\right)<{TTC}^{*}\right)=\left\{\begin{array}{c} 1,TTC\left(t\right)<{TTC}^{*}\\ 0,TTC\left(t\right)\geq {TTC}^{*} \end{array}\right.$	
			Here,	${TTC}^{*}$: TTC threshold
				$TTC\left(t\right)$: TTC at time t
TIT	Cumulative risk exposure below the TTC threshold	TTC	$\int_{t_{1}}^{t_{2}}{(TTC}^{*}-TTC(t))dt$ $TTC\left(t\right)<{TTC}^{*}$	
Jerk	Rate of change of acceleration over time	longitudinal_acceleration, lateral_acceleration	$j_{long}\left(t_{k}\right)=\frac{a_{long}\left(t_{k}\right)-a_{long}\left(t_{k-1}\right)}{t_{k}-t_{k-1}};$ $j_{lat}\left(t_{k}\right)=\frac{a_{lat}\left(t_{k}\right)-a_{lat}\left(t_{k-1}\right)}{t_{k}-t_{k-1}}$; $j_{total}\left(t_{k}\right)=\sqrt{j_{long}{\left(t_{k}\right)}^{2}+j_{lat}{\left(t_{k}\right)}^{2}}$	
			Here,	$a_{long}\left(t_{k}\right)$: longitudinal acceleration at time $t_{k}$
				$a_{lat}\left(t_{k}\right)$: lateral acceleration at time
				$j_{long}\left(t_{k}\right)$: longitudinal jerk
				$j_{lat}\left(t_{k}\right)$: lateral jerk
				$j_{total}\left(t_{k}\right)$: total jerk

**Table 5:** Summary of analysis criteria and thresholds.

Analysis Step	Criterion or Threshold	Purpose
Event reconstruction	Same object class and temporal gap ≤ 2 s within the same or spatially adjacent camera views	To reconstruct vehicle–VRU interaction events from frame-level detections
Driving-event selection	Average vehicle speed ≥ 5 km/h	To exclude stopped or low-speed waiting events for which TTC is difficult to interpret
DBT calculation	Time from initial SVM detection to first brake activation	To quantify the conditional brake response latency after initial detection
TTC calculation	Estimated distance divided by approaching relative speed; $v_{rel}>0$	To calculate the event-level time margin under an approaching condition
TTC validity	Positive TTC values within the predefined analysis range; 0 < TTC ≤ 15 s	To exclude non-approaching or unstable TTC values
TET/TIT threshold	TTC ≤ 3 s	To define below-threshold risk exposure intervals
Event-level TTC	5th percentile TTC within each event	To represent high-risk intervals while reducing frame-level noise
Jerk indicators	Mean and 95th percentile jerk within each event	To characterize overall and high-intensity vehicle dynamic responses
Statistical comparison	Mann–Whitney U test, Cliff’s delta, Holm correction	To compare event-level distributions and effect sizes between risk zones

**Table 6:** Interpretation criteria for the Cliff’s delta effect size.

Effect Size Level	Absolute Cliff’s Delta $\left|d\right|$	Interpretation
Negligible	$\left|d\right|<0.147$	Very small or trivial difference
Small	$0.147\leq \left|d\right|<0.330$	Small difference
Medium	$0.330\leq \left|d\right|<0.474$	Moderate difference
Large	$\left|d\right|>0.474$	Large difference

**Table 7:** Summary of the data.

Date	Location	Driving Time (h)	Distance (km)	Detection (Count)
29 April 2026	Gangseo	4.29	101.58	74,912
30 April 2026	Dongjak	2.72	61.53	66,368
11 May 2026	Gangnam	1.53	24.44	54,670
12 May 2026	Gangnam	3.03	36.8	51,922
13 May 2026	Gangnam	2.01	37.2	82,023
14 May 2026	Gangnam	2.16	33.94	59,385
Total		15.74	295.49	389,280

**Table 8:** Distribution of detected object counts by location.

Location	Pedestrian	Bicycle	Motorcycle	PM	Total
Gangseo	59,616	5145	10,140	11	74,912
Dongjak	54,729	3053	8584	2	66,368
Gangnam	205,198	20,812	18,659	3331	248,000
Total	319,543	29,010	37,383	3344	389,280

**Table 9:** Summary of TTC, TET, and TIT by date and risk zone.

Location		Zone	Mean TTC (s)	Median TTC (s)	Mean Duration (s)	Mean TET (s)	Mean TIT ($s^{2}$)	TIT /TET (s)
Gangseo		Danger	1.42	0.72	0.79	0.25	0.45	1.85
		Warning	1.74	0.94	1.75	0.47	0.81	1.57
Dongjak		Danger	1.65	0.83	1.3	0.34	0.59	1.76
		Warning	1.80	0.96	1.89	0.5	0.84	1.61
Gangnam	Day 1	Danger	2.16	1.35	1.50	0.31	0.52	1.57
		Warning	3.88	3.50	2.42	0.14	0.23	1.46
	Day 2	Danger	1.72	0.99	1.18	0.20	0.35	1.71
		Warning	2.67	1.79	1.35	0.14	0.23	1.43
	Day 3	Danger	1.44	0.80	1.40	0.38	0.65	1.68
		Warning	4.86	4.23	1.44	0.09	0.15	1.48
	Day 4	Danger	1.5	0.81	1.30	0.25	0.41	1.84
		Warning	2.94	1.84	2.74	0.28	0.45	1.47

**Table 10:** Summary of jerk indicators by date and risk zone.

Location		Zone	Mean Total Jerk	p95 Total Jerk	Mean Longitudinal Jerk	Mean Lateral Jerk	p95 Longitudinal Jerk	p95 Lateral Jerk
Gangseo		Danger	0.192	0.649	0.135	0.082	0.463	0.233
		Warning	0.211	0.813	0.159	0.075	0.601	0.311
Dongjak		Danger	0.233	0.758	0.172	0.102	0.571	0.257
		Warning	0.250	0.968	0.192	0.082	0.726	0.381
Gangnam	Day 1	Danger	0.225	0.77	0.185	0.121	0.586	0.347
		Warning	0.242	0.936	0.205	0.100	0.963	0.390
	Day 2	Danger	0.225	0.64	0.164	0.094	0.507	0.317
		Warning	0.220	0.909	0.173	0.087	0.689	0.347
	Day 3	Danger	0.214	0.621	0.146	0.095	0.417	0.311
		Warning	0.225	0.888	0.154	0.090	0.627	0.335
	Day 4	Danger	0.167	0.532	0.118	0.077	0.377	0.224
		Warning	0.217	0.877	0.155	0.079	0.589	0.325

**Table 11:** Overall statistical comparison of pedestrian safety indicators by risk zone.

Indicator	N (Danger)	N (Warning)	Mean (Danger)	Mean (Warning)	Median (Danger)	Median (Warning)	Adjusted *p*-Value	Cliff’s Delta	Effect Size
DBT	85	266	0.9906	1.0128	0.69	0.8235	0.8703	−0.0118	Negligible
TTC	1889	4206	1.8626	2.8193	0.8267	1.4864	<0.001	−0.199	Small
TET	1995	4714	0.2471	0.2265	0.0000	0.0000	<0.001	0.0939	Negligible
TIT	1995	4714	0.4239	0.3812	0.0000	0.0000	<0.001	0.0994	Negligible
TIT/TET	1804	3289	1.7557	1.5212	1.8409	1.5596	<0.001	0.1882	Small
Mean total Jerk	1416	3153	0.2086	0.2235	0.1328	0.1899	<0.001	−0.097	Negligible
P95 total Jerk	1416	3153	0.675	0.8859	0.3122	0.6561	<0.001	−0.1537	Small
Mean long. Jerk	1416	3153	0.1545	0.1696	0.0654	0.1271	<0.001	−0.1203	Negligible
P95 long. Jerk	1416	3153	0.4963	0.6597	0.1356	0.3867	<0.001	−0.1584	Small
Mean lat. Jerk	1416	3153	0.0858	0.0896	0.0413	0.0618	<0.001	−0.0943	Negligible
P95 lat. Jerk	1416	3153	0.2667	0.3438	0.0958	0.2012	<0.001	−0.1388	Negligible

**Table 12:** Trend analysis of p95 total jerk during repeated driving in Gangnam.

Zone	Coefficient of Day_No	*p*-Value	Trend Interpretation
Danger	−0.0711	0.0204	Exploratory decreasing trend
Warning	−0.0019	0.9367	No significant trend

## Data Availability

The datasets presented in this article are not readily available because they are part of an ongoing study and subject to institutional restrictions. Requests to access the datasets should be directed to the corresponding author, Jong-Hoon Kim (kjh4004@kict.re.kr).

## Abbreviations

The following abbreviations are used in this manuscript:

ADAS	Advanced driver-assistance system
DBT	Detection-to-braking time
FCW	Forward collision warning
FHWA	Federal Highway Administration
KEIT	Korea Planning and Evaluation Institute of Industrial Technology
OBD	On-board diagnostics
PET	Post-encroachment time
SSMs	Surrogate safety measures
SVM	Surround view monitoring
TET	Time-exposed TTC
TIT	Time-integrated TTC
TTC	Time to collision
VRU	Vulnerable road user
